# Combined association of physical activity and diet with C-reactive protein among smokers

**DOI:** 10.1186/s40200-015-0181-y

**Published:** 2015-06-24

**Authors:** Paul D. Loprinzi, Jerome F. Walker

**Affiliations:** Department of Health, Exercise Science, and Recreation Management, Center for Health Behavior Research, School of Applied Sciences, The University of Mississippi, 215 Turner Center, University, MS 38677 USA; Department of Respiratory Therapy, Bellarmine University, Louisville, KY USA

**Keywords:** Accelerometry, Dietary behavior, Epidemiology, Nicotine, Smoking

## Abstract

**Background:**

In the general population, both physical activity and dietary behavior are independently associated with less systemic inflammation, with this relationship less examined among smokers. To our knowledge, no study has examined the *combined* association of both physical activity and dietary behavior on systemic inflammation among daily smokers, which was the purpose of this study.

**Methods:**

Data from the 2003–2006 NHANES were employed. 810 adult smokers provided C-reactive protein data (CRP; a marker of inflammation), and sufficient physical activity (accelerometry) and dietary data (healthy eating index).

**Results:**

The fully adjusted model showed that participants meeting physical activity guidelines and eating a healthy diet (β = −0.34, *p* = 0.03) had lower CRP levels when compared to those not engaging in these health behaviors, but only having one health behavior was not a significant predictor of CRP (β = −0.19, *p* = 0.14).

**Conclusions:**

Smokers engaging in regular physical activity while consuming a healthy diet demonstrate lower CRP levels than their counterparts. When taken together, these behaviors may mitigate inflammation associated with various chronic diseases, which is of particular importance as very few smokers successfully quit smoking.

## Introduction

Smoking triggers chronic immunologic responses that upregulate biomarkers associated with inflammation and multi-organ disease, including plasma C-reactive protein (CRP) [1–3]. This, coupled with the relatively low smoking cessation rate (~3 %) among U.S. adults underscores the importance of daily smokers engaging in health-enhancing behaviors such as physical activity and healthy eating [4]. Yet physical activity and healthy eating are less prevalent in daily smokers and inversely associate with systemic inflammation [5–8].

Low-grade systemic inflammation has been associated with physical inactivity when using elevated CRP as a biomarker [9]. Conversely, 6–35 % lower CRP levels have been reported in response to various intensities of physical activity, independent of baseline CRP levels and body composition [10]. Moreover, the benefit of physical activity has been observed among diverse populations, ranging from women participating in 16 weeks of aerobic exercise [11] to Multiple Sclerosis patients walking at a comfortable pace 5 h weekly for 24 weeks [12], and among current or former smokers with COPD [13]. The mechanism(s) through which physical activity positively influences specific inflammatory activity (e.g., CRP) associated with cardiovascular disease remains unclear. One plausible explanation is the indirect effect of physical activity on CRP reduction through changes in body weight. Adipocytes have been shown to synthesize cytokines (IL-1, TNF-alpha) which are involved in the production of CRP [14]. By reducing adipose mass, physical activity could decrease cytokine production, which, in turn, could decrease CRP concentration. Physical activity has also been suggested to mitigate inflammation by improving insulin resistance, as inflammatory markers are typically raised in insulin-resistant individuals [15]. Another possible mechanism through which physical activity may decrease inflammation is by improving endothelial function. Activated endothelial cells can increase the production of IL-1, IL-6 and adhesion molecules, thereby inducing inflammation [16]. Regular physical activity has been shown to improve endothelial function by preserving nitric oxide availability [17] and by reducing peripheral inflammatory markers (e.g., adhesion molecules) associated with endothelial dysfunction [18].

In addition to physical inactivity, systemic inflammation associates with an unhealthy diet [9]. Dietary fiber intake is reported to be inversely related to CRP [19] among multiple populations, including adolescents [9], young and middle aged adults [20], low income urban inhabitants [21], and overweight/obese, and post-menopausal women [22]. Lowered CRP has been associated with dietary improvements among overweight and obese men and women that also increased their physical activity [23]. Consumption of a healthy diet, including adequate fruits and vegetables, has been shown to reduce inflammation both in *in vitro* and animal models. Quercetin reduced TNF-alpha and CRP in streptozotocin-induced diabetic rats [24], which were accompanied by attenuated blood pressure and vasoconstriction. Hesperitin, a common flavonoid in citrus fruits, reduced inflammatory cytokines TNF-α and IL-1β in diabetic rat retina [25].

In theory, regular physical activity coupled with healthy eating should have an additive inverse effect on inflammation among smokers, but we are aware of no study examining this possibility. As a result, the primary purpose of this study of adult U.S. smokers was to examine if regular physical activity coupled with healthy eating lowers CRP to a greater extent than these individual behaviors. Here the focus was daily smokers because few are able to quit and compared to non-smokers, they are less active and eat less healthily.

## Methods

### Study design and participant

Data were restricted to the 2003–2006 National Health and Nutrition Examination Survey (NHANES) cycles because these are the only present cycles with objectively measured physical activity data (i.e., accelerometry). The NHANES is an ongoing survey conducted by the Centers for Disease Control and Prevention that uses a representative sample of non-institutionalized United States civilians selected by a complex, multistage, stratified, clustered probability design. The multistage design consists of 4 stages, including the identification of counties, segments (city blocks), random selection of households within the segments, and random selection of individuals within the households. In the 2003–2006 cycle, participants were sampled across 15 different U.S. geographic areas during each 2-year cycle. Participants were interviewed in their homes and then subsequently examined in a mobile examination center (MEC) by NHANES personnel. NHANES study procedures were approved by the National Center for Health Statistics ethics review board, with informed consent obtained from all participants prior to data collection.

In the 2003–2006 NHANES, 1345 adults (≥20 year of age) were daily smokers (i.e., self-reported smoking every day) and provided data on dietary behavior and all the covariates. After excluding those with missing or insufficient accelerometry data (i.e., < 4 days of 10+ hrs/day of monitoring data), 810 participants remained, which constituted the analytic sample. When comparing the 535 participants who were excluded due to missing or insufficient accelerometry data to the 810 analyzed participants, those excluded were less likely to be male (62 % vs. 55 %; *p* = 0.007), less likely to have emphysema (4.3 % vs. 2.2 %, *p* = 0.04), less likely to have diabetes (9.0 % vs. 5.6 %, *p* = 0.02), had a lower healthy eating index score (46.1 vs. 44.8, *p* = 0.02), were younger (46.4 vs. 39.8 years; *p* < 0.001), had a lower poverty-to-income ratio (2.4 vs. 2.0, *p* < 0.001), and had a higher CRP level (0.42 vs. 0.55 mg/dL, *p* = 0.01). These are unweighted estimates.

### Measurement of physical activity

While attending the MEC, participants were instructed to wear an ActiGraph 7164 accelerometer during all activities, except water-based activities and while sleeping. The accelerometer measured the frequency, intensity, and duration of physical activity by generating an activity count proportional to the measured acceleration. The accelerometer output is digitized using an analog-to-digital converter, and once digitized, the signal passes through a digital filter that detects accelerations ranging from 0.05 to 2.00 g in magnitude with frequency responses ranging from 0.25 to 2.5 Hz to filter motion outside normal human movement. The filtered signal is then rectified and summed over a pre-determined epoch period. After the activity count is sorted into an epoch, it is stored in the internal memory and the integrator is reset to zero. Detailed information on the ActiGraph accelerometer can be found elsewhere [26]. Estimates for moderate-to-vigorous physical activity were summarized in 1-min time intervals. Activity counts greater than or equal to 2020 were classified as moderate-to-vigorous physical activity intensity [27]. For the analyses described here, and to represent habitual physical activity patterns, only those participants with activity patterns for at least 4 days of 10 or more hours per day of monitoring data were included in the analyses [27]. To determine the amount of time the monitor was worn, nonwear was defined by a period of a minimum of 60 consecutive minutes of zero activity counts, with the allowance of 1–2 min of activity counts between 0 and 100 [27].

Participants were classified as sufficiently active if they engaged in at least 150 min a week of moderate-to-vigorous physical activity. SAS (version 9.2) was used to reduce the accelerometry data using code provided the National Cancer Institute. Using the SAS code, the average time each participant spent per day in physical activity was analyzed from valid individual data.

### Measurement of dietary behavior/healthy eating index

Two 24-h recall assessments of food and fluid intake were collected during participant visits to a MEC. To capture intake on all days of the week, the 24-h recalls were collected on every day of the week. The dietary interviewers used the Dietary Data Collection (DDC) system, which is an automated standardized interactive dietary interview and coding system. The Healthy Eating Index (HEI) 2005 was developed by the USDA as an indicator of dietary quality. The HEI is comprised of 12 components (total fruit; whole fruit; total vegetable; dark green, orange vegetable and legumes; total grain; whole grain; milk; meat and beans; oil; saturated fats; sodium; and calories from solid fats, alcoholic beverages, and added sugars) with each component individually scored, with a maximum total score of 100. A higher score reflects closer adherence to the dietary guidelines for Americans. The HEI was derived for each of the 24-h recall days using the MyPyramid Equivalents Database and following the methods and SAS code established by the USDA Center for Nutrition Policy and Promotion [28–31].

Using the average of the two-day HEI scores, participants at or above the 60th percentile (i.e. top 40 %) of HEI scores in the population were categorized as adhering to the dietary guidelines or consuming a healthy diet [32].

### Calculation of summed positive health characteristics

Given that 2 positive health characteristics were assessed (i.e., sufficiently active and healthy diet), participants were classified as having 0–2 positive health characteristics by summing the number of health characteristics they had.

### Measurement of high sensitivity CRP

During examination at the MEC, blood samples were obtained from participants. High sensitivity CRP concentration was quantified using latex-enhanced nephelometry, and reported in mg/dL to the nearest hundredth (0.01). The coefficients of variation (CV) by lot ranged from 3.1 % to 9.9 %.

### Measurement of covariates

Based on previous research demonstrating an association with physical activity, diet and CRP, covariates included age, gender, race-ethnicity, poverty-to-income ratio (PIR), DXA-determined body fat percent, cotinine, mean arterial pressure, self-reported cholesterol medication use, self-reported smoking cessation agent use (e.g., bupropion), and physician diagnosis of chronic bronchitis, emphysema, diabetes, cancer, and coronary artery disease.

As a measure of socioeconomic status, PIR was assessed (a value < 1 was considered below the poverty threshold). The PIR is calculated by dividing the family income by the poverty guidelines, which is specific to the family size, year assessed, and state of residence. Total body fat percent was estimated from whole-body DXA scans using the Hologic QDR 4500A fan beam x-ray bone densitometer (Hologic, Inc, Bedford, Massachusetts). Examination of the DXA data showed that missing data for total body fat percent demonstrated a systematic, non-random pattern; therefore, only assessing participants with measured data for total body fat percent would lead to biased results. Therefore, missing DXA values for total body fat percent were imputed by NCHS personnel using multiple imputation procedures (i.e., sequential regression multivariate imputation) [33]. Ultimately 5 total body fat percent values for each participant were generated. The average of these 5 values was used to define DXA-determined body fat percent. Normal weight was defined as 5–20 % for men and 8–30 % for women; overweight was 21–24 % for males and 31–36 % for females; and obese was > 24 % for males and >36 % for females [34].

Serum cotinine, a biological marker of passive/active smoking, was measured by an isotope dilution-high performance liquid chromatography/atmospheric pressure chemical ionization tandem mass spectrometry. Blood pressure was measured 3 or 4 times, and the average mean arterial pressure ([diastolic blood pressure × 2) + systolic blood pressure]/3) was calculated.

### Data analysis

Statistical analyses (Stata, version 12.0, College Station, TX) accounted for the complex survey design used in NHANES. To account for oversampling, non-response, non-coverage, and to provide nationally representative estimates, all analyses included the use of survey sample weights, stratum and primary sampling units. Sample weights were created for the combined NHANES cycles following analytical guidelines for the continuous NHANES.

Multivariable linear regression analysis was used to examine the association between the number of positive health behaviors (independent variable) and log-transformed CRP (due to non-normality). Three models were computed; an unadjusted model (Model 1), a minimally adjusted model (Model 2) which included covariates age, gender, race-ethnicity, PIR and weight status, and a fully adjusted model (Model 3) which included the same covariates in model 2 plus cotinine, emphysema, bronchitis, diabetes, cancer, coronary artery disease, mean arterial pressure, smoking cessation agent use, and cholesterol medication use.

Following these multivariable linear regression models, a multivariable logistic regression analysis was computed examining the association between numbers of positive health behaviors (independent variable) and having an elevated CRP level (i.e., > 0.3 mg/dL vs. ≤ 0.3 mg/dL).

In addition to examining whether lower CRP levels was present among those engaging in both health behaviors, additive and multiplicative interaction for physical activity and diet was assessed. As described by Kalilani and Atashili [35], and broadly speaking, additive interaction exists when the joint effect of the risk factors differs from the *sum* of the effects of the individual factors. Additive interaction was tested by calculating the relative excess risk due to interaction (RERI), the attributable proportion due to interaction (AP), and the synergy index (S), using the methods described by Andersson and colleagues [36]. The RERI can be interpreted as the risk that is additional to the risk that is expected on the basis of the addition ORs under exposure; AP is interpreted as the proportion of the condition that is due to interaction among persons with both exposures; and S is interpreted as the excess risk from exposure to both exposures when there is interaction relative to the risk from exposure without interaction [37].

To calculate these parameters, a logistic regression was computed with elevated CRP serving as the outcome variable. The regression coefficients from the physical activity and dietary variables, along with having both health behaviors, were entered into the Excel sheet developed by Andersson and colleagues [36]. Then, the covariance matrix of the coefficients of the logistic model was computed with the appropriate covariances from the covariance matrix entered into the Excel sheet to provide an estimate of RERI, AP, and S. Statistical significance was evaluated with 95 % confidence intervals with AP and RERI departing from 0, and S departing from 1.0 indicating additive interaction. Multiplicative interaction was tested by including a cross-product term for meeting physical activity guidelines and eating a healthy diet along with the main effect terms for each in the regression model. Statistical significance was set at *p* ≤ 0.05.

## Results

Table 1 shows the weighted smoker characteristics across the number of positive health behaviors. The weighted mean number of cigarettes smoked per day was 17.2 (95 % CI: 16.1–18.3). Participants with more health behaviors were younger, of male gender, had a higher PIR, lower cotinine level, less likely to have emphysema, chronic bronchitis, and stroke, and had lower CRP levels.

**Table 1:** Weighted characteristics of U.S. smokers across number of positive health behaviors, 2003–2006 NHANES (*n* = 810)

	Mean/Proportion (SE)			
	Number of positive health behaviors (Sufficiently active and healthy diet)			
Variable	0 (*n* = 270)	1 (*n* = 436)	2 (*n* = 104)	*P*-value^†^
Demographic				
Age, yrs	43.3 (1.1)	42.1 (0.6)	40.4 (1.1)	0.01
Gender, %				
Male	47.7 (3.2)	62.7 (2.8)	63.4 (6.5)	0.01
Race-Ethnicity, %				0.41
Mexican American	2.6 (0.6)	5.4 (1.3)	6.3 (1.9)	
Non-Hispanic White	78.5 (3.0)	77.0 (2.8)	76.5 (4.5)	
Non-Hispanic Black	11.5 (2.1)	10.4 (2.0)	8.6 (2.0)	
Other Race	7.1 (1.9)	7.0 (1.1)	8.4 (3.7)	
Poverty-to-income ratio	2.3 (0.1)	2.9 (0.1)	3.2 (0.1)	<0.001
Body Fat, %^‡^				0.27
Normal Weight	10.2 (2.4)	15.8 (2.2)	14.3 (4.3)	
Overweight	24.5 (3.4)	16.6 (1.9)	21.3 (5.4)	
Obese	65.2 (3.7)	67.5 (3.4)	64.3 (5.6)	
Cotinine, ng/mL	270.1 (7.7)	241.4 (7.5)	222.0 (14.5)	0.004
Cholesterol Medication, %				
Yes	11.0 (2.1)	9.5 (1.6)	4.4 (2.7)	0.25
Smoking Cessation Agent, %				
Yes	3.1 (1.4)	2.1 (0.9)	3.0 (2.0)	0.82
Comorbidities/Health				
Emphysema, %				
Yes	9.3 (2.4)	2.1 (0.6)	0.4 (0.4)	<0.001
Chronic Bronchitis, %				
Yes	16.0 (2.1)	8.9 (1.6)	4.4 (1.8)	0.004
Diabetes, %				
Yes	6.5 (1.4)	4.5 (1.0)	2.9 (1.3)	0.19
Coronary Artery Disease, %				
Yes	1.7 (0.7)	2.9 (0.9)	1.7 (1.6)	0.58
Stroke, %				
Yes	4.5 (1.3)	1.5 (0.6)	0	0.01
Cancer, %				
Yes	8.5 (1.9)	5.3 (1.0)	7.9 (2.9)	0.38
Mean Arterial Pressure, mmHg	87.3 (0.8)	87.4 (0.7)	86.8 (1.4)	0.72
Biomarker				
C-Reactive Protein (not log-transformed), mg/dL	0.51 (0.1)	0.34 (0.03)	0.32 (0.06)	0.01
Physical Activity and Diet				
MVPA, min/day	9.2 (0.3)	28.9 (1.2)	42.0 (1.7)	<0.001
AHEI	39.7 (0.3)	47.2 (0.6)	53.7 (0.4)	<0.001

*MVPA* Moderate-to-vigorous physical activity; *AHEI* Average Healthy Eating Index

^†^For continuous variables (e.g., age), a linear regression was used to make comparisons across the age groups, with 0 positive health behaviors serving as the referent group and the corresponding *p*-value comparing 2 health behaviors to 0 health behaviors. For categorical variables (e.g., gender), a design-based likelihood ratio test was used

^‡^Normal weight = 5–20 % for males and 8–30 % for females

Overweight = 21–24 % for males and 31–36 % for females

Obese = > 24 % for males and >36 % for females

Table 2 displays the weighted multivariable association between number of positive health behaviors and log-transformed CRP. The fully adjusted model (Model 3) showed that participants with both health behaviors (β = −0.34, *p* = 0.03) had lower CRP levels, but only having one health behavior was not a significant predictor of CRP (β = −0.19, *p* = 0.14).

**Table 2:** Weighted association between multiple health behaviors and C-reactive protein among U.S. daily smokers

	β (SE) Δ in log C-reactive protein (mg/dL)^†^					
Health behaviors	Model 1	P	Model 2	P	Model 3	P
1 vs. 0	−0.26 (0.1)	0.06	−0.19 (0.1)	0.13	−0.19 (0.1)	0.14
2 vs. 0	−0.48 (0.1)	0.01	−0.34 (0.1)	0.05	−0.34 (0.1)	0.03
Covariates						
Age, 1 year older			0.01 (0.003)	0.003	0.01 (0.004)	0.07
Female vs. Male			0.20 (0.1)	0.01	0.23 (0.1)	0.003
Race-Ethnicity						
Mexican American vs. White			0.01 (0.1)	0.96	0.0001 (0.1)	1.0
Black vs. White			0.25 (0.1)	0.01	0.20 (0.1)	0.06
Other vs. White			0.15 (0.1)	0.30	0.15 (0.1)	0.28
PIR, 1 unit higher			−0.05 (0.02)	0.03	−0.05 (0.02)	0.04
Body Fat						
Overweight vs. Normal Weight			0.22 (0.1)	0.18	0.22 (0.1)	0.20
Obese vs. Normal Weight			1.06 (0.1)	<0.001	0.98 (0.1)	<0.001
Cotinine, 1 ng/mL higher					−0.00002 (0.001)	0.96
Emphysema vs. No Emphysema					−0.19 (0.3)	0.45
Bronchitis vs. No Bronchitis					0.15 (0.1)	0.30
Diabetes vs. No Diabetes					0.23 (0.2)	0.26
Cancer vs. No Cancer					0.22 (0.2)	0.28
Coronary Artery Disease vs. No Coronary Artery Disease					0.15 (0.2)	0.53
Mean Arterial Pressure, 1 mmHg higher					0.01 (0.003)	0.001
Smoking Cessation Agent vs. No Agent					−0.38 (0.4)	0.39
Cholesterol Medication vs. Not on Cholesterol Medication					0.03 (0.1)	0.81

^†^3 models were computed

Model 1 is an unadjusted model

Model 2 (minimally adjusted) controlled from age, gender, race-ethnicity, poverty level, and body fat percent

Model 3 (fully adjusted) controlled for same covariates in Model 2 plus cotinine, emphysema, bronchitis, diabetes, cancer, coronary artery disease, mean arterial blood pressure, use of smoking cessation agents, and use cholesterol medication

Additional secondary analyses were computed with elevated CRP (>0.3 mg/dL) serving as the outcome variable. After complete adjustment (same covariates in Model 3 of Table 2), having 2 (vs. 0) health behaviors (OR = 0.53, *p* = 0.05), but not 1 (vs. 0) health behavior (OR = 0.79, *p* = 0.33), was associated with lower odds of having an elevated CRP level (not shown in tabular format).

Further analyses were computed to examine the independent association of each health behavior on log-transformed CRP. After complete adjustment, and with both healthy diet and meeting physical activity guidelines entered into the model, meeting physical activity guidelines (β = −0.22, *p* = 0.03) but not healthy diet (β = −0.13, *p* = 0.22) predicted log-transformed CRP (not shown in tabular format). Similarly, and with regard to elevated CRP, after complete adjustment, meeting physical activity guidelines (OR = 0.66, *p* = 0.03) but not healthy diet (OR = 0.83, *p* = 0.54) predicted elevated CRP (not shown in tabular format).

There was no evidence of statistical interaction. The completely adjusted multiplicative interaction model was not significant for log-transformed CRP (β = 0.03, *p* = 0.90) or elevated CRP (OR = 0.84, *p* = 0.70). Similarly, the additive interaction results were not significant; RERI = −0.18 (95 % CI: −1.07-0.71), AP = −0.10 (95 % CI: −0.63-0.41), and S = 0.78 (95 % CI: 0.28-2.19). Similarly, there was no multiplicative interaction of obesity with the summed health behavior variable on CRP (β = 0.05, *p* = 0.58). Lastly, additional analyses were computed to see if the inclusion of other covariates (e.g., marital status, years smoked, alcohol behavior or statin/aspirin use) significantly or appreciably changed the results; findings were similar so other covariates were not included in the models. Also, when we re-computed the results after excluding those with low (<800 kcals/day for men and < 600 kcals/day for women) or high (>5000 kcals/day for men and > 4000 kcals/day for women) caloric intake, the results were unchanged (data not shown).

## Discussion

The primary purpose of this study was to examine the additive association of physical activity and diet on inflammation among U.S. adult daily smokers. After adjustment, physical activity, but not diet, was independently associated with CRP. Both sufficient physical activity and eating a healthy diet were additive in predicting CRP to a greater extent than physical activity alone, but there was no additive or multiplicative *interaction,* suggesting that the joint effect of physical activity and diet was not greater than the sum of their individual effects.

As discussed in the introduction section, research has demonstrated independent effects of physical activity and diet on CRP among the general population. To our knowledge, the present study is the first to examine the combined association (additive effect) of overall healthy eating and physical activity on CRP among smokers. Although not examining a combined association, our findings are similar to others that have demonstrated an association between physical activity and inflammation among smokers [38].

Nicotine dependence is exceedingly problematic and, arguably, deserves priority over inactivity or poor dietary habits. Most smokers would like to quit; only 3 % of those attempting are successful at 6 months [39], leaving most subject to ongoing lung, cardiovascular, and oncologic risks. It was on this basis that we chose to examine daily smokers with a focus on effects of improving their physical activity and diet—examining for possibly favorable additive or additive interaction effects on anti-inflammatory responses using CRP as a biomarker. Further prospective studies are needed to confirm our findings, which are that maintaining even a modest level of physical activity and a healthy diet may additively slow the progression of comorbid cardiovascular and metabolic disorders by reducing inflammation among daily smokers. By extension, while changing both physical activity and diet is optimal, starting with physical activity first may be an alternative. This sequence provides a possible antecedent basis for smoking cessation. There is a growing literature supporting the neurocognitive benefits focused on physical activity as a starting point [40], with several meta-analytic reviews showing a positive association between physical activity and cognitive function, particularly executive functioning [41]. Dysregulation among 3 core brain networks has been associated with nicotine withdrawal symptoms, namely, executive function, self-referential thinking, and orientation toward external versus internal stimuli. Accordingly, reduced executive function impairs top-down cognitive control to resist cravings to smoke [42]. Physical activity participation among smokers may help to facilitate smoking cessation via increased executive function. There is also evidence to suggest that physical activity-induced increases in executive function may also facilitate changes in dietary behavior [40].

### Limitations and strengths

The main limitation of this study was the cross-sectional study design, which precludes any ability to ascertain temporal precedence, and thus, infer any causal effects. Although participants were drawn from a nationally representative sample, another limitation is that the excluded sample was different than the analyzed sample due to missing data; in particular, excluded participants had a higher CRP and a lower HEI, which suggests that our health behavior-CRP associations may have been underestimated. Also, it is possible that accelerometry reactivity may be a concern among smokers; however, we have a limited understanding of whether this occurs in this population. Major strengths of this study include the study design employed by NHANES, utilizing objective measures of physical activity (not prone to recall bias and social desirability bias) and smoking, examining the novel combined association of diet and physical activity on CRP among smokers, and employing additive interaction statistical models.

## Conclusion

In conclusion, smokers engaging in regular physical activity while consuming a healthy diet demonstrate lower CRP levels than their counterparts engaging in one or none of these behaviors. When adopted concurrently, and if confirmed by prospective and experimental work, these behaviors may mitigate various chronic diseases associated with systemic inflammation. Further research is needed on possible additive and salutary effects of physical activity and diet on executive function as an antecedent to smoking cessation. Although at the current moment it is reasonable to consider CRP as a pro-inflammatory mediator of various chronic diseases, future clinical trials using CRP inhibitors are needed to confirm a causative role between CRP and cardiovascular disease [43, 44]. Lastly, future studies are also encouraged to measure additional biomarkers (e.g., TNF-α, IL-1β, and IL-6; not available in the NHANES) involved in inflammation that may be influenced by physical activity, dietary behavior and smoking.
